# Refractory anaphylactic shock complicated by acute right heart failure during anesthetic induction in a patient with a left ventricular assist device: a case report

**DOI:** 10.1186/s40981-026-00864-6

**Published:** 2026-05-25

**Authors:** Shohei Kaneko, Sojiro Matsumoto, Taiga Ichinomiya, Ushio Higashijima, Motohiro Sekino, Tetsuya Hara

**Affiliations:** Department of Anesthesiology and Intensive Care Medicine, Nagasaki University Graduate School of Biomedical Sciences, 1-7-1 Sakamoto, Nagasaki, 852-8501 Japan

**Keywords:** Adrenaline, Anaphylactic shock, Epinephrine, Left ventricular assist device, Refractory shock, Remimazolam, Right heart failure

## Abstract

**Background:**

Anaphylaxis in patients with a left ventricular assist device (LVAD) may critically reduce LVAD flow, particularly when the right ventricular function is impaired.

**Case presentation:**

A 51-year-old man with a HeartMate III LVAD developed bronchospasm, profound hypotension, and decreased LVAD flow during anesthetic induction for endoscopic sinus surgery. The shock remained refractory despite epinephrine boluses and continuous infusions of epinephrine, norepinephrine, and vasopressin. Transesophageal echocardiography revealed severe right ventricular dilatation, leftward septal shift, and reduced left ventricular size, findings consistent with acute right heart failure (RHF). Treatment was escalated to include dobutamine, olprinone, and inhaled nitric oxide, achieving recovery of blood pressure and LVAD flow. Elevated serum tryptase supported the diagnosis of anaphylaxis, and a positive intradermal test identified remimazolam as the cause.

**Conclusions:**

Successful anaphylaxis management in LVAD recipients may require rapid recognition and treatment of acute RHF in addition to standard treatment including epinephrine.

## Background

In recent years, the number of left ventricular assist device (LVAD) recipients undergoing non-cardiac surgery has increased [1]. Adequate preload and right ventricular function are essential for maintaining LVAD flow, which is sensitive to afterload [1–3]. Because right ventricular dysfunction is common in LVAD recipients [1], perioperative hemodynamic instability may be poorly tolerated and can significantly compromise LVAD support.

Anaphylaxis is an important cause of perioperative hemodynamic instability and can rapidly progress to life-threatening shock if not promptly recognized and treated [4]. Epinephrine is the mainstay of anaphylaxis treatment; however, some cases are refractory to standard therapy [4, 5]. Acute right heart failure (RHF) may contribute to this refractory state, particularly when bronchospasm causes hypoxemia and hypercapnia, thereby increasing right ventricular afterload [5]. This mechanism may be especially relevant in LVAD recipients with impaired baseline right ventricular function. Herein, we describe an LVAD recipient who developed anaphylactic shock refractory to standard therapy during anesthetic induction, in whom echocardiography revealed findings consistent with acute RHF.

## Case presentation

The patient was a 51-year-old man (height, 162 cm; weight, 72 kg) with a history of dilated cardiomyopathy and severe heart failure, managed with an LVAD (HeartMate III; Abbott Laboratories, Chicago, IL, USA) implanted three years previously. A cardiac resynchronization therapy defibrillator (CRT-D) was implanted nine years previously for dilated cardiomyopathy. He was scheduled to undergo elective endoscopic sinus surgery for right maxillary sinusitis in preparation for planned future heart transplantation and subsequent immunosuppression. His preoperative medications included bisoprolol 1.25 mg/day, sacubitril/valsartan 200 mg/day, amiodarone 100 mg/day, sotalol 80 mg/day, aspirin 100 mg/day, and warfarin 1 mg/day. The patient had no known drug allergies or history of asthma.

**Fig. 1:**
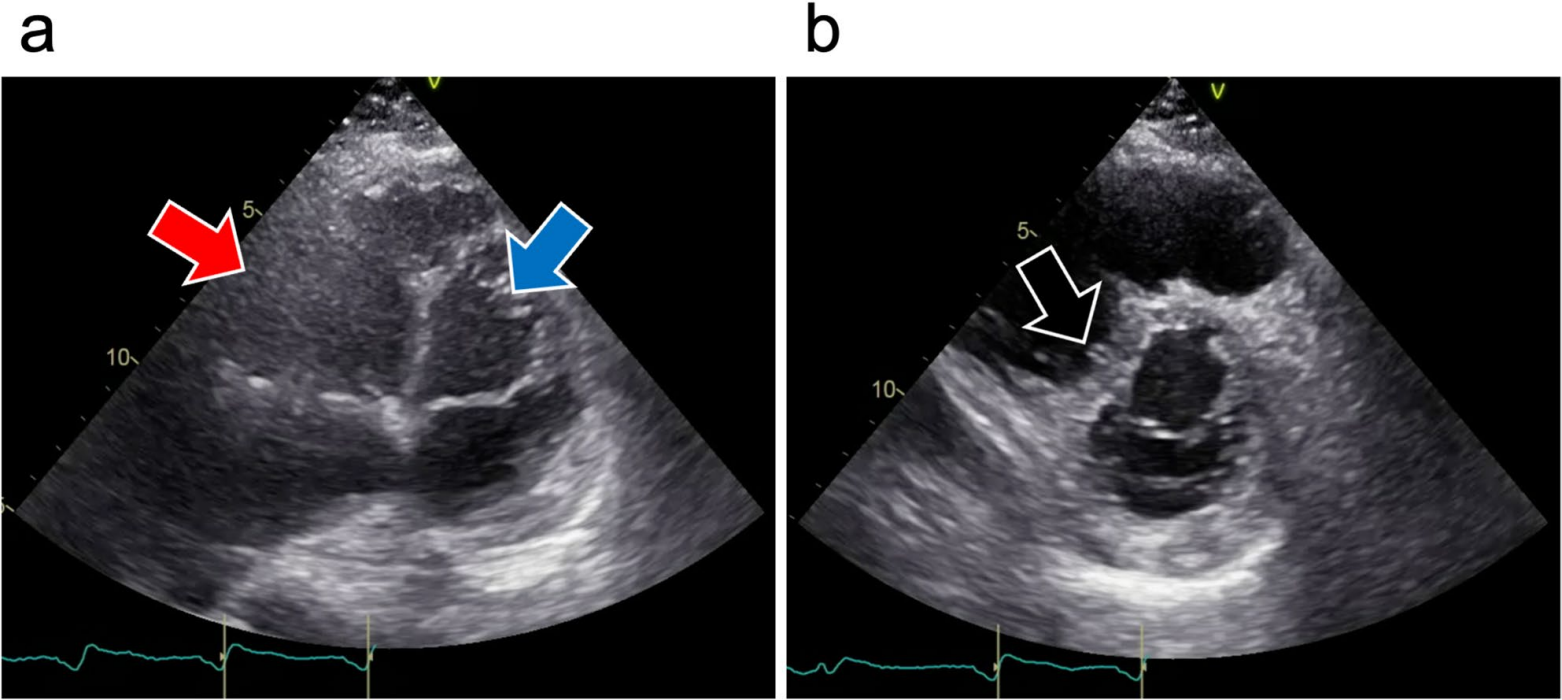
Preoperative transthoracic echocardiography. **a**: Apical four-chamber view demonstrating right ventricular dilation (red arrow) relative to the left ventricle (blue arrow). **b**: Left ventricular short-axis view at the mitral valve level showing flattening of the interventricular septum (black arrow)

Preoperative cardiac evaluation consisted of a 12-lead electrocardiogram (ECG) and transthoracic echocardiography (TTE). The ECG demonstrated a paced rhythm at 90 beats/min (pacemaker set to VVIR, 90/min). TTE confirmed severe left ventricular dysfunction (ejection fraction 15%; end-diastolic/end-systolic diameters 49/45 mm) and indicated impaired right ventricular function (fractional area change 22%). Morphologically, TTE demonstrated right ventricular dilation on apical view (Fig. 1a) and septal flattening on the left ventricular short-axis view (Fig. 1b), consistent with right ventricular pressure and volume overload.

In the operating room, standard monitoring, including bispectral index and regional cerebral oxygen saturation monitors, was established, along with invasive arterial and central venous pressure monitoring. Anesthesia was induced with remimazolam 3 mg, remifentanil 1 µg/kg/min, and rocuronium 70 mg, while norepinephrine was continuously infused at 0.05 µg/kg/min. Remimazolam was continued via infusion at 1 mg/kg/h. One minute after induction, difficulty with mask ventilation developed. Simultaneously, the mean arterial pressure (MAP) plummeted from 80 mmHg to approximately 20 mmHg, and LVAD output decreased from 3.8 L/min to approximately 1.5 L/min. Endotracheal intubation was performed two minutes after the event, but a peak inspiratory pressure (PIP) of 34 cmH₂O was required to deliver a tidal volume of 500 mL. Despite two bolus doses of 0.2 mg of phenylephrine, the MAP only rose into the 30-mmHg range.

**Fig. 2:**
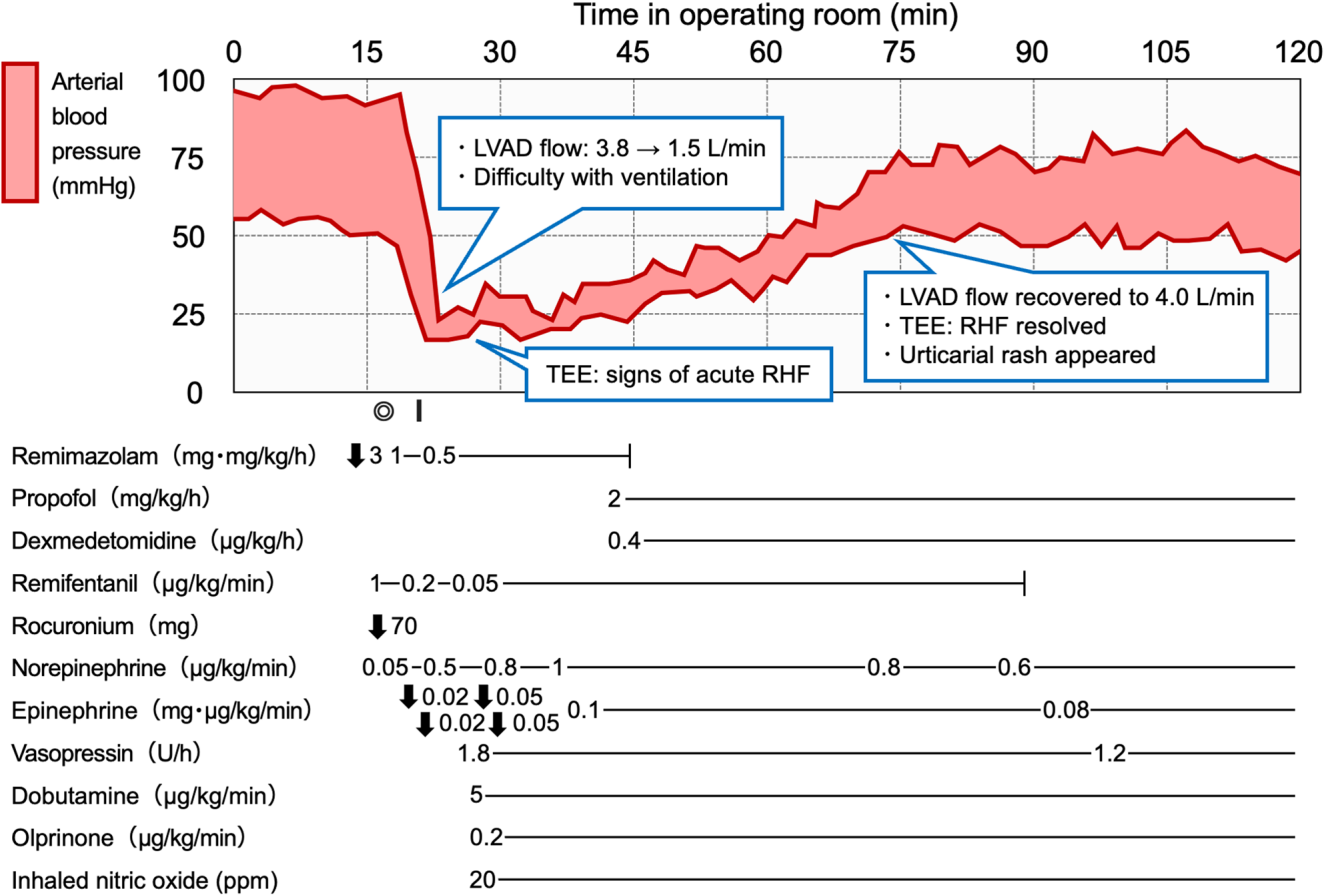
Clinical course during anesthetic induction and resuscitation. Time-course graph showing changes in arterial blood pressure during anesthetic induction and resuscitation. Available left ventricular assist device flow values are shown at the corresponding time points. Double circles indicate the start of oxygen administration, and vertical bars indicate tracheal intubation. LVAD, left ventricular assist device; TEE, transesophageal echocardiography; RHF, right heart failure; ppm, parts per million

Perioperative anaphylaxis related to induction agents was suspected, and 0.02 mg of epinephrine was consequently administered. Epinephrine was repeated at doses of 0.02 mg, 0.05 mg, and 0.05 mg and then a continuous infusion at 0.1 µg/kg/min was initiated. Norepinephrine was titrated up to 1 µg/kg/min, and vasopressin was initiated at 1.8 U/h. Although airway resistance decreased, allowing ventilation with a PIP of 20 cmH₂O, the MAP remained in the 40-mmHg range, indicating a poor hemodynamic response. The clinical course during anesthetic induction and resuscitation is shown in Fig. 2.

**Fig. 3:**
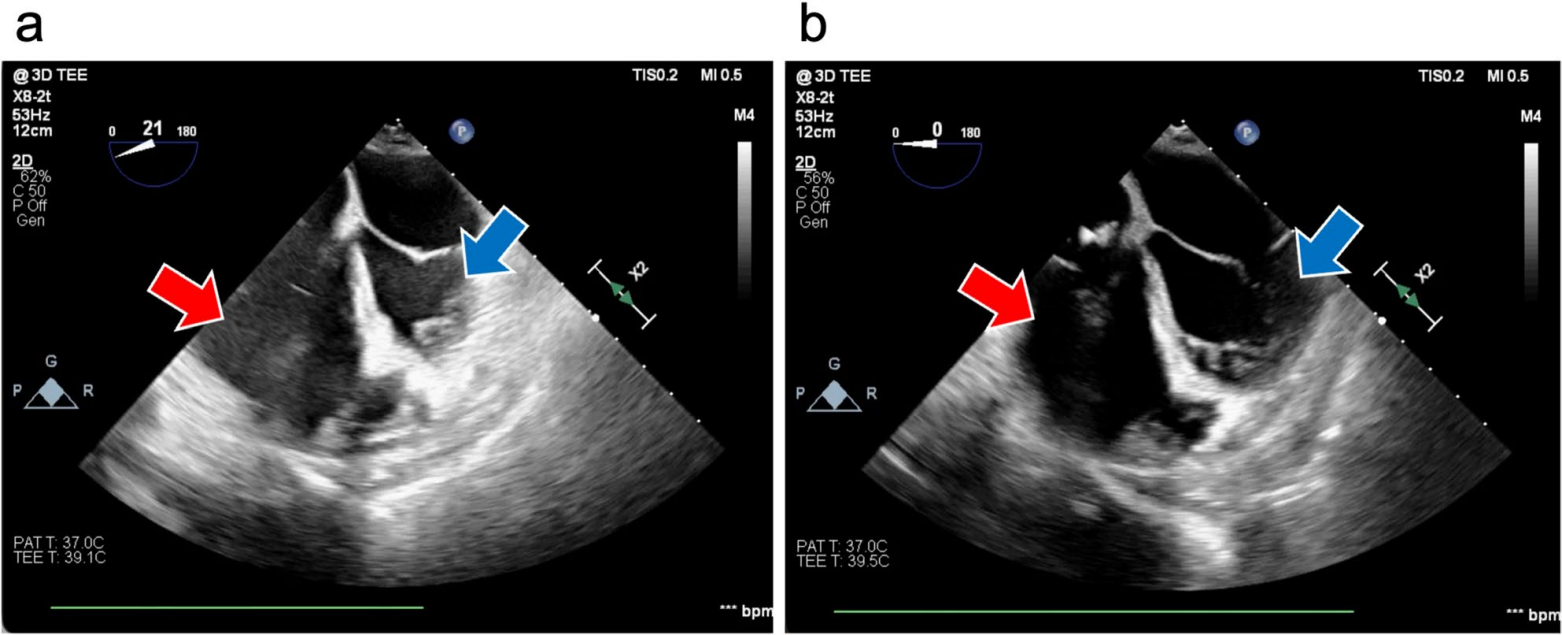
Mid-esophageal four-chamber views on transesophageal echocardiography. **a**: During anaphylactic shock refractory to standard therapy, the interventricular septum was displaced leftward, with right ventricular dilatation (red arrow) and reduced left ventricular size (blue arrow), findings consistent with severe right heart failure. **b**: Echocardiographic findings 30 min after initiating dobutamine, olprinone, and inhaled nitric oxide therapy; the right ventricular dilatation is reduced (red arrow), and left ventricular filling has been achieved (blue arrow), confirming resolution of acute right heart failure

Transesophageal echocardiography (TEE) was performed to assess cardiac function and demonstrated leftward displacement of the interventricular septum, right ventricular dilatation, and reduced left ventricular size (Fig. 3a). These findings were consistent with severe RHF. Based on the TEE findings, treatment was escalated to RHF-directed therapy, including dobutamine at 5 µg/kg/min, olprinone, a phosphodiesterase Ⅲ inhibitor, at 0.2 µg/kg/min, and inhaled nitric oxide (iNO). Over the following 30 min, the MAP gradually increased to 60 mmHg, and LVAD flow recovered to 4.0 L/min. Follow-up TEE confirmed resolution of the acute RHF findings (Fig. 3b). Throughout this course, regional cerebral oxygen saturation remained between 70% and 80%, with no decrease from baseline. As the hemodynamics improved, an urticarial rash appeared on the patient’s anterior chest. Remimazolam and remifentanil were discontinued and replaced with propofol and dexmedetomidine. The planned surgery was cancelled, and the patient was transferred to the intensive care unit (ICU) for further management. The total fluid administration during the operating room stay was 1,600 mL, and urine output was 100 mL.

**Fig. 4:**
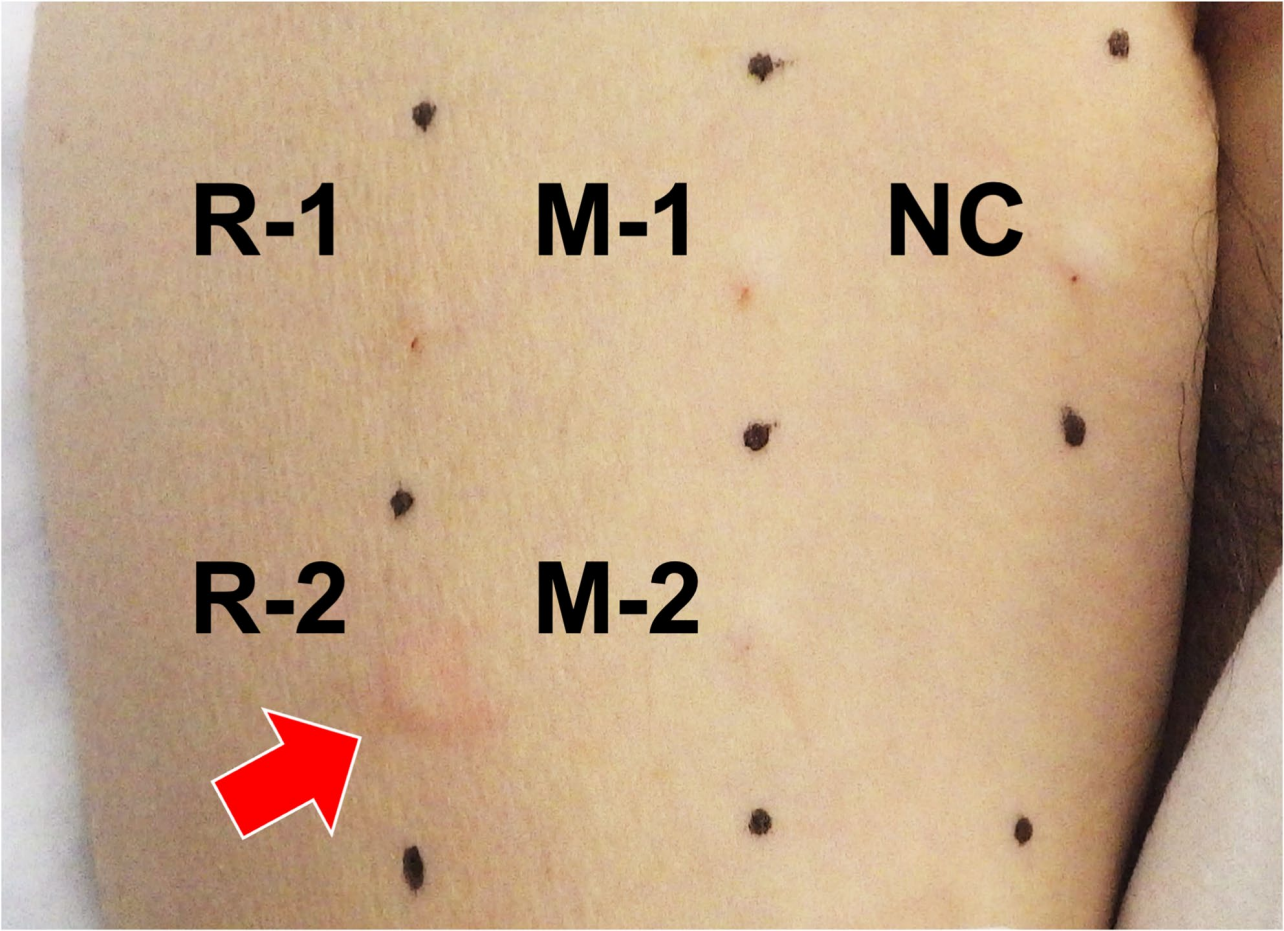
Photograph of skin tests for anaphylaxis. Intradermal test showing reactions to remimazolam, midazolam, and the negative control (saline). R-1 and R-2 represent remimazolam solution (5 mg/mL), diluted 1:10,000 (0.5 μg/mL) and 1:1,000 (5 μg/mL) in saline, respectively. M-1 and M-2 represent midazolam solution (5 mg/mL), diluted 1:10,000 (0.5 μg/mL) and 1:1,000 (5 μg/mL) in saline, respectively. The red arrow indicates a positive response in R-2

In the ICU, the patient’s condition stabilized, allowing for the gradual withdrawal of circulatory support agents; the continuous epinephrine infusion was discontinued 12 h after ICU admission. Extubation was successful the day after the anaphylactic event, and iNO therapy was discontinued two hours after extubation. The patient exhibited no neurological deficits. Serum tryptase levels measured during the anaphylactic event were elevated (34.7 µg/L at 1 h and 11.5 µg/L at 24 h after onset). Four weeks after the anaphylactic event, skin tests were performed to identify the allergen. Four drugs were tested: the suspected agents remimazolam, remifentanil, and rocuronium, along with midazolam, a benzodiazepine sedative similar to remimazolam. For intradermal testing, a positive response was defined according to criteria described in the Japanese Society of Allergology Skin Test Guide 2025: a mean erythema diameter of ≥ 20 mm or a mean wheal diameter of ≥ 9 mm, with each mean diameter calculated as the mean of the longest diameter and its orthogonal diameter [6]. The results showed a positive intradermal response to remimazolam (Fig. 4). These findings support remimazolam as the cause of anaphylaxis, and we communicated this information to the patient’s heart transplant center. Subsequently, the patient underwent the planned endoscopic sinus surgery under general anesthesia with propofol, remifentanil, and rocuronium without any adverse events. Ten months after the anaphylactic event, the patient successfully underwent heart transplantation.

The patient provided written informed consent for publication of this case report.

## Discussion

We report a case of remimazolam-induced anaphylactic shock complicated by acute severe RHF in an LVAD recipient. In LVAD recipients, effective circulatory support depends on adequate preload and right ventricular function [1–3]. Because right ventricular dysfunction is common in this population,^1^ acute increases in right ventricular afterload can critically compromise LVAD flow and systemic hemodynamics. In the present case, anaphylaxis was accompanied by severe hypotension, bronchospasm, and a marked reduction in LVAD flow, while transesophageal echocardiography revealed findings consistent with acute severe RHF, indicating that acute RHF contributed to the refractory shock.

Anaphylaxis can impair right ventricular function through several mechanisms. Bronchospasm can lead to hypoxemia and hypercapnia, increasing right ventricular afterload [5]. In addition, mediators released during anaphylaxis may directly increase pulmonary vascular resistance (PVR) [5], while marked systemic vasodilation may reduce preload and coronary perfusion despite LVAD support [2]. Epinephrine remains the mainstay of anaphylaxis treatment [4]; however, its alpha-1 adrenergic effects may further increase PVR under certain conditions, particularly when acidosis is present during resuscitation [5]. In LVAD recipients with impaired baseline right ventricular function, these factors can promote acute RHF and contribute to refractory shock.

In the present case, severe hypotension persisted despite escalation of vasopressor therapy, including high-dose epinephrine. TEE played an important diagnostic role by demonstrating right ventricular dilatation, leftward septal shift, and reduced left ventricular size, which are findings consistent with acute severe RHF. Based on this assessment, treatment was escalated to include dobutamine, olprinone, and iNO, in parallel with ongoing standard anaphylaxis therapy. Dobutamine and olprinone were administered to support right ventricular contractility [1, 3], whereas iNO was used to selectively dilate the pulmonary vasculature, thereby reducing right ventricular afterload [7]. The subsequent recovery of blood pressure and LVAD flow, together with resolution of the echocardiographic abnormalities, indicates that this RHF-directed strategy was effective. Vasopressin may also have been a reasonable adjunct in this setting because it is recommended for epinephrine-refractory anaphylaxis [4], and has minimal effect on PVR, which may be advantageous in LVAD recipients [2, 3]. 

To our knowledge, only two prior case reports have described anaphylaxis in patients with an LVAD, both of which occurred during LVAD-related cardiac surgery [8, 9]. The first reported severe anaphylaxis to chlorhexidine during LVAD implantation and again during a heart transplantation attempt; in both episodes, hemodynamics improved rapidly after epinephrine administration [8]. The second described refractory anaphylactic shock following aprotinin administration during heart transplantation, although the mechanism of refractory shock was not explored [9]. Detailed reports of anaphylaxis in LVAD recipients during non-cardiac surgery, as in the present case, remain extremely limited. The present report is clinically relevant as it suggests that acute RHF may be an important contributor to refractory anaphylactic shock in this population and highlights the diagnostic value of echocardiography. However, because this is a single-case report, the generalizability of this management approach remains uncertain.

In summary, we report a case of remimazolam-induced anaphylactic shock in a patient with an LVAD complicated by acute RHF. This case demonstrates that severe RHF can occur during anaphylaxis in LVAD recipients, thereby emphasizing the diagnostic value of echocardiography. Successful anaphylaxis management in LVAD recipients may require not only standard treatment, including epinephrine, but also rapid recognition and treatment of acute RHF.

## Data Availability

The datasets generated and analyzed during the current study are available from the corresponding author upon reasonable request.

## Abbreviations

CRT-D: Cardiac resynchronization therapy defibrillator
ECG: Electrocardiogram
iNO: Inhaled nitric oxide
LVAD: Left ventricular assist device
MAP: Mean arterial pressure
PIP: Peak inspiratory pressure
PVR: Pulmonary vascular resistance
RHF: Right heart failure
TEE: Transesophageal echocardiography
TTE: Transthoracic echocardiography
